# Impact of HIV-1 Backbone on Neutralization Sensitivity: Neutralization Profiles of Heterologous Envelope Glycoproteins Expressed in Native Subtype C and CRF01_AE Backbone

**DOI:** 10.1371/journal.pone.0076104

**Published:** 2013-11-29

**Authors:** Agnès-Laurence Chenine, Lindsay Wieczorek, Eric Sanders-Buell, Maggie Wesberry, Teresa Towle, Devin M. Pillis, Sebastian Molnar, Robert McLinden, Tara Edmonds, Ivan Hirsch, Robert O’Connell, Francine E. McCutchan, David C. Montefiori, Christina Ochsenbauer, John C. Kappes, Jerome H. Kim, Victoria R. Polonis, Sodsai Tovanabutra

**Affiliations:** 1 The Henry M. Jackson Foundation, Bethesda, Maryland, United States of America; 2 Military HIV Research Program, Silver Spring, Maryland, United States of America; 3 University of Alabama, Birmingham, Birmingham, Alabama, United States of America; 4 Walter Reed Army Institute of Research, Silver Spring, Maryland, United States of America; 5 Bill & Melinda Gates Foundation, Seattle, WA, United States of America; 6 Duke University, Durham, North Carolina, United States of America; 7 Inserm UMR891, Centre de Recherche en Cancérologie de Marseille, Marseille, France; Chinese Academy of Medical Sciences, China

## Abstract

Standardized assays to assess vaccine and antiviral drug efficacy are critical for the development of protective HIV-1 vaccines and drugs. These immune assays will be advanced by the development of standardized viral stocks, such as HIV-1 infectious molecular clones (IMC), that i) express a reporter gene, ii) are representative of globally diverse subtypes and iii) are engineered to easily exchange envelope (env) genes for expression of sequences of interest. Thus far, a subtype B IMC backbone expressing *Renilla* luciferase (LucR), and into which the ectodomain of heterologous *env* coding sequences can be expressed has been successfully developed but as execution of HIV-1 vaccine efficacy trials shifts increasingly to non-subtype B epidemics (Southern African and Southeast Asia), non-subtype B HIV-1 reagents are needed to support vaccine development. Here we describe two IMCs derived from subtypes C and CRF01_AE HIV-1 primary isolates expressing LucR (IMC.LucR) that were engineered to express heterologous gp160 Envs. 18 constructs expressing various subtypes C and CRF01_AE Envs, mostly acute, in subtype-matched and –unmatched HIV backbones were tested for functionality and neutralization sensitivity. Our results suggest a possible effect of non-*env* HIV-1 genes on the interaction of Env and neutralizing antibodies and highlight the need to generate a library of IMCs representative of the HIV-1 subtype spectrum to be used as standardized neutralization assay reagents for assessing HIV-1 vaccine efficacy.

## Introduction

 The HIV-1 envelope (Env) glycoproteins are produced as a 160 kDa polyprotein that is subsequently processed to yield virion-associated, trimeric complexes of non-covalently associated gp120-gp41 heterodimers [1,2]. The surface subunit, gp120, is responsible for the specific binding of virions to target cells; gp41, the transmembrane subunit, mediates fusion of viral and cellular membranes [3]. Neutralizing antibodies (NAbs) can block virus entry by binding Env and inhibiting attachment or conformational changes required for fusion [4–7]. Env structural studies have primarily focused on gp120 and the extracellular domain of gp41 (i.e. ectodomain), where the small panel of known broadly NAbs bind [8]; the cytoplasmic tail (CT) of gp41 (endodomain) is considered to be entirely contained inside the virion [9,10] and consequently is thought not to be targeted by the host immune response. However, studies have suggested a more complex role of the gp41 endodomain [11,12], showing neutralization of HIV-1 by Abs directed to an epitope in CT of gp41 [13–15]. Mutations in the CT have been shown to affect the conformation of gp120 ectodomain [16–18], and more recently, Durham et al suggested that the CT regulates the conformation of Env at the cell surface and control epitope exposure through T cell virological synapses [19]. These results emphasize the importance of the gp41 endodomain and the rationale to express and study the complete gp160 derived from primary isolates.

These issues intersect practically in the viral reagents that are commonly used in HIV-1 neutralization assays, which form an important component in the evaluation of candidate HIV vaccines. In support of HIV vaccine development, intensive collaborative efforts have now yielded reference panels of HIV-1 Envs representative of the worldwide viral genetic diversity and have standardized neutralization assay systems [20–24]. Most of these data measured neutralization of Env-pseudotyped viruses in TZM-bl cells [21–24]. More recently, a new HIV-1 Env expression vector has been developed, allowing for multiple rounds of replication and easy read-out in HIV-1 natural target cells [25–27]. However both assays relied solely on T-cell line-adapted B strains to express HIV-1 Env and generate functional HIV-1 viral stocks [28,29]. As execution of HIV-1 vaccine efficacy trials shifts increasingly to non-subtype B epidemics (Southern African and Southeast Asia), new HIV-1 reagents representing the full genetic diversity of non-B HIV-1 subtypes are needed to support vaccine development targeting non-B HIV strains. 

 To bridge this gap, we have developed new IMC.LucR HIV-1 constructs derived from native subtype C and CRF01_AE strains. We further modified these vectors to express subtype C and CRF01_AE full-length gp160 Env in subtype-matched and -mismatched HIV-1 backbones. Replication of these constructs was tested in different cell types. Monoclonal antibody (mAb) and plasma-mediated neutralization sensitivity of various HIV-1 Envs were compared when expressed in these two non-subtype B HIV-1 backbones. We observed variation in neutralization sensitivity when Env is expressed in subtype-matched vs –mismatched HIV backbone. These findings indicate that non-*env* genes may play an important role in subtype-specific neutralization sensitivity. Our results suggest that a library of HIV-1 backbones representative of viral diversity should be generated and their usage for analysis of immune responses carefully weighed.

## Materials & Methods

For clarity, all the primers used in this study are listed in Table S1.

### Cells

 The TZM-bl cell line was obtained through the NIH AIDS Research and Reference Reagent Program, Division of AIDS, NIAID, NIH: TZM-bl from Dr. John C. Kappes, Dr. Xiaoyun Wu and Tranzyme Inc. [30]. The 293T/17 Human kidney cell line (CRL-11268) was obtained from the American Type Culture Collection (ATCC). Both cell lines were maintained in Dulbecco’s modified Eagle’s medium (DMEM) supplemented with 15% heat-inactivated fetal bovine serum (PAA laboratories), 1% penicillin/streptomycin (Gibco/BRL). The A3R5.7 cell line (McLinden et al., manuscript in preparation) was maintained in RPMI 1640 growth medium supplemented with 15% heat-inactivated fetal bovine serum (PAA laboratories), 1% L-Glutamine and 1% penicillin/streptomycin (Gibco/BRL) and 600 µg/ml (acitive) geneticin (G418, Gibco/BRL). Human PBMC (peripheral blood mononuclear cells) were isolated by Ficoll gradient from buffy coats obtained from healthy HIV-1 seronegative donors. Aliquots of 1x10^7^ PBMC were kept frozen at -150C until needed. After thawing, PBMC were stimulated with 5 g/ml of phytohemagglutinin (PHA)-P and cultured in RPMI 1640 growth medium supplemented with 15% heat-inactivated fetal bovine serum (PAA laboratories), 1% L-Glutamine, 1% Penicillin/Streptomycin (Gibco/BRL) and 20U/ml recombinant human IL-2 (Roche Applied Sciences)

### Generation of CRF01_ AE and subtype C HIV-1 IMC.LucR

**Figure 1:**
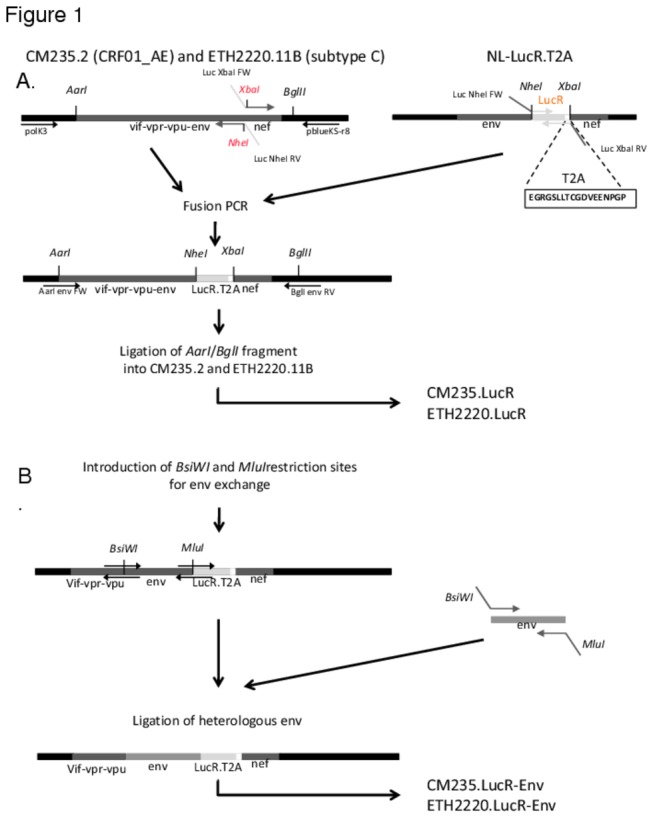
IMC.LucR construction. Schematic representation of the construction of (A) primary isolate-derived IMC.LucR and (B) chimeric IMC.LucR-Env.

 To insert the cassette LucR.T2A from pNL-LucR.T2A [25] into AE.CM235.2 [31] and C.ETH2220.11B [32], we chose unique enzyme restriction sites encompassing the entire env and nef gene sequences; AarI cuts at position 5062 in pol/vif, and BglI, which cuts in CM235.2 at position 9628, and in ETH2220.11B at the position 9630 (Figure 1A). AarI is present in 93% of the HIV-1 sequences that we screened in the Los Alamos database (www.hiv.lanl.gov), making it an ideal molecular tool for the cloning of LucR.T2A into all HIV-1 subtypes.

 We first amplified three overlapping fragments, obtained using the following cycles: 1 cycle at 94C for 2 min, 27 cycles at 94C for 15s, 55C for 30s, 68C for 5 min, and a final extension step at 68C for 15 min (Platinum Taq, Invitrogen). Env of CM235.2 and ETH2220.11B were amplified using the forward primer polk3 and the reverse primer LucNheIRV, the cassette LucR.T2A in NL-LucR.T2A [25] was amplified with LucNheIFW and LucXbaIRV, and finally nef was amplified with LucXbaIFW and pblueKS-r8. PCR products obtained were gel purified with S.N.A.P. UV-free gel purification kit (Invitrogen) and finally quantified on 0.8% EtBr-agarose gel using Gene Ruler DNA ladder mix (Fermentas).

 The three resulting fragments were then subjected to the double-step DNA amplification approach described in Shevchuck et al. [33]. The first step was without primers (1 cycle at 94C for 60s, and 12 cycles at 94C for 15s, 68C for 10 min), and the second step was with the primers AarIENVFW and BglIENVRV (1 cycle at 94C for 60s, and 30 cycles at 94C for 15s, 68C for 11min with 5s increment and a final cycle of 72C for 15 min) (Advantage GC, Clontech). 

### Generation of Chimeric AE- and C-IMC.LucR-Env expressing heterologous env genes

 In order to identify the enzyme restriction sites to generate chimeric IMCs for heterologous Env expression, HIV-1 sequences from all subtypes retrieved from Los Alamos database were examined (www.hiv.lanl.gov). We found two enzyme restriction sites BsiWI and MluI, which are mostly absent in HIV-1; respectively, 99.1% and 98.7% of the HIV-1 sequences do not display these restriction sites. Hence, the BsiWI site was inserted at position 6202 in HXB2, in a conserved sequence (90% conserved in the CRF01_AE and subtype C vpu sequences) of the cytoplasmic region of vpu, by replacing a Glutamate with a Valine. MluI site was added after the env stop codon (Figure 1B). Briefly, using QuickChange II site-directed Mutagenesis kit (Agilent Technologies), CM235.LucR was amplified first with the complementary primer pair A/E_BsiWIvpuFW and A/E_BsiWIvpuRV, and ETH2220.LucR DNA with C-BsiWIvpuRV and C_BsiWIvpuFW using the following cycles: 1 cycle at 95C for 30s and 18 cycles at 95C for 30s, 55C for 60s, 68C for 15min. The MluI restriction site was then introduced following the same protocol into BsiWI clones using the following complementary primer pairs: for CM235.LucR, MluI_EFW and MluI_ERV and for ETH2220.LucR, MluI_CFW and MluI_CRV. 

 The insertion of the restriction site BsiWI in the vpu gene generated an amino acid change (position 6202 in HXB2). To confirm the functionality of our chimeric IMC.LucR with the modified-vpu, we titered transfection-derived viruses with and without the amino acid change in vpu in TZM-bl cells, and observed no differences (Figure S1). 

### Cloning

 Purified inserts and backbones CM235.2 and ETH2220.11B were digested with the required restriction enzymes; AarI, BglI, MluI, and BsiWI (Fermentas/Thermoscientific). The clones with the right insert were determined by visualizing on 0.8% EtBr agarose gel and further confirmed by sequencing using two primers situated in the envelope to differentiate between parental plasmids and clones carrying the right insert. The clones obtained were then screened for infectivity in TZM-bl cells. The infectious ones were selected and purified via PureYield Plasmid Maxiprep System (Promega), and sequenced to confirm that the whole env gene was fully inserted. 

### Virus stock production

 To produce IMC stocks, a 75 cm^2^ flask was seeded at a density of 5x10^6^ 293T/17 cells one-day prior to transfection. 16 g proviral DNA and 72 l of FuGENE 6 transfection reagent (Roche) were used according to the manufacturer’s protocol. Viral supernatants were harvested 48-60 hrs post-transfection, filtered through 0.2 m pore size filters, aliquoted into 0.5 ml or 1 ml and stored at -150C. The main reason for the extensive usage of some subtype B IMCs such as pNL4-3 or pSG3 as HIV-1 backbone is their robust infectivity due to laboratory adaptation. The two non-B IMCs that we are describing here were isolated with limited “ex vivo” cell culture and exhibit modest infectivity in some cases, but we believe these are more physiologically relevant reagents. In order to overcome this issue, when needed, virus stocks were concentrated after the filtration step using Amicon Ultra-15 Centrifugal Filter Units with Ultracel-100 membrane (Millipore) according to the manufacturer’s protocol. An aliquot before centrifugation was always kept to measure the titer increase obtained with this procedure.

 Pseudovirus stocks were produced similarly through co-transfecting 8 g Env expression plasmid and 8 g Env-deficient HIV-1 backbone vector (pSG3env) [7,30]. Primary isolates were produced by propagation in 5.0x10^6^ PBMC. Culture supernatants were harvested 7 days post-infection and stored as 1ml-aliquots at -150C. 

### Virus functionality

 Relative viral infectivity was assessed for each virus stock by viral titration (endpoint dilution assay) to determine TCID_50_ (50% tissue culture infectious dose) for each virus in the different cell types, removing the need to standardize viral input according to p24 content. We use a cut-off value of 3-times over background (uninfected cells) relative light units (RLU) for Firefly Luciferase (FF) as described elsewhere. For Renilla Luciferase (LucR), we determine the cut-off as being 3x[(average of negative wells)+3SD]. In the TZM-bl reporter cell line [29], 96-well black plates were seeded at a density of 2x10^4^ cells per well and infected using duplicate or triplicate wells with 4-fold virus dilutions, in the presence of DEAE-Dextran (20 g/ml, Sigma) [34]. After a 48-hour incubation, FF activity was measured using the Bright-Glo Luciferase Assay System for pseudoviruses and IMC, and Dual-Glo Luciferase Assay System for IMC.LucR (Promega); FF and LucR activity can be both measured simultaneously in the same infected cells using the Dual-Glo Luciferase Assay System (Promega). 

 IMC.LucR infectivity was also tested on A3R5.7 cells and PBMC. 96-well black plates were seeded at a cell density of 1x10^5^ with (A3R5.7 cells) and without (PBMC) the addition of of 25 g/ml DEAE-Dextran. The same virus dilutions were used on all cell types and LucR activity was quantified 94 hours post-infection (Renilla-Glo Luciferase Assay System). RLU were collected on a Victor X light Luminescence counter (Perkin-Elmer) by using an exposure time of 0.1 s/well. 

### Neutralization assay

 Inhibition of HIV-1 infection with different mAbs and sera was analyzed in TZM-bl cells and PBMC. TZM-bl neutralization assays were performed as previously described [35]. Neutralization was measured as a reduction in FF RLU in the presence of serial antibody/serum dilutions. PBMC neutralization assays were performed essentially as previously described [25,26]. Neutralization was measured as the reduction in LucR RLU in the presence of serial antibody/serum dilutions. For both assay platforms, viral input used was normalized to a dilution for each virus expected to produce a level of RLU at least 10-times above cut off value (as described above). The reciprocal titers were derived through averaging values from two independent assays for which the IC_50_ values were within 3-fold range. The following reagents were tested: 4E10, 2G12, B12, 2F5 (Polymun), sCD4 (Progenics Pharmaceuticals), subtypes B, CRF01_AE and C pooled sera/plasmas (previously described [35]), AIP3441 and AIP3254 (individual CRF01_AE HIV+ plasma samples from Thailand, Dr. Nicos Karasavvas), 1026, 1027 and 1028 (individual subtype B HIV+ plasma samples from the US collected under the RV149 IRB approved protocol).

#### Human Use

HIV-1 negative PBMC were collected by leukapheresis from a single donor under the Walter Reed Army Institute of Research IRB approved clinical study RV229C (WRAIR#1386) protocol. Plasma samples 1026, 1027 and 1028 were collected under the Walter Reed Army Institute of Research IRB approved clinical study RV149 (WRAIR#1011) protocol. Written consent was obtained under a procedure approved by WRAIR institutional review board (IRB).

### Data Analysis

 DNA sequences were assembled and analyzed using Sequencher version 5.0 (Genecodes Inc., Ann Arbor, MI). Statistical analyses were performed and Wilcoxon non-parametric tests were used to assess whether there were significant differences in the neutralization sensitivities by reagent and between constructs from different subtypes 

## Results

### Subtype C and CRF01_AE IMC.LucR mediate productive infection

 The two native non-subtype B IMC.LucR were generated as described in Figure 1A; the cassette LucR.T2A expressed in the proviral vector NL-LucR.T2A [25] was successfully inserted into the CRF01_AE IMC, CM235.2 [31] and the subtype C IMC, ETH2220.11B [32] generating the CRF01_AE and subtype C IMC.LucR viruses, CM235.LucR and ETH2220.LucR, respectively. Next, we engineered unique restrictions sites into these constructs to allow for exchange of different heterologous full-length HIV-1 env into either CRF01_AE or subtype C genetic backgrounds (Figure 1B). For this study, we focused our attention on the cloning of subtypes C and CRF01_AE envs, from both acute and chronic stages of HIV-1 infection (as described in Table 1); however, this cloning strategy can be used to express Envs from any subtype.

**Table 1:** List of subtype C and CRF01_AE chimeric IMC.LucR.

**Envelope (gp160)**	**Env Subtype**	**Env expressed in backbone subtype C ETH2220.11B**	**Env expressed in backbone CRF01_AE CM235.2**	**Env Fiebig Stage**	**Env Accession number** ^1^
356272	CRF01_AE	**√**	**√**	I/II	JN944654
427299	CRF01_AE	**√**	**√**	I/II	JN944655
620345	CRF01_AE	**√**	**√**	I/II	JN944656
644039	CRF01_AE	**√**	**√**	I/II	JN944657
703357	CRF01_AE	**√**	**√**	I/II	JN944658
R2184.04	CRF01_AE	**√**	**√**	VI and beyond	JN944665
CM235.2	CRF01_AE	**√**	**√ (homologous)**	VI and beyond	JN944662
6980v0	C	**√**	**√**	I/II	PCT/US12/35026^2^
6838v1	C	**√**	**√**	I/II	HM215341
6838v7	C	**√**	**√**	VI and beyond	Pending^3^
ETH2220.11B	C	**√ (homologous)**	**√**	VI and beyond	U46016

^1^ Sequences to be found on Genbank http://www.ncbi.nlm.nih.gov/genbank/

^2^ International Application Number for filing patent in the US

^3^ Sequences being submitted

**Figure 2:**
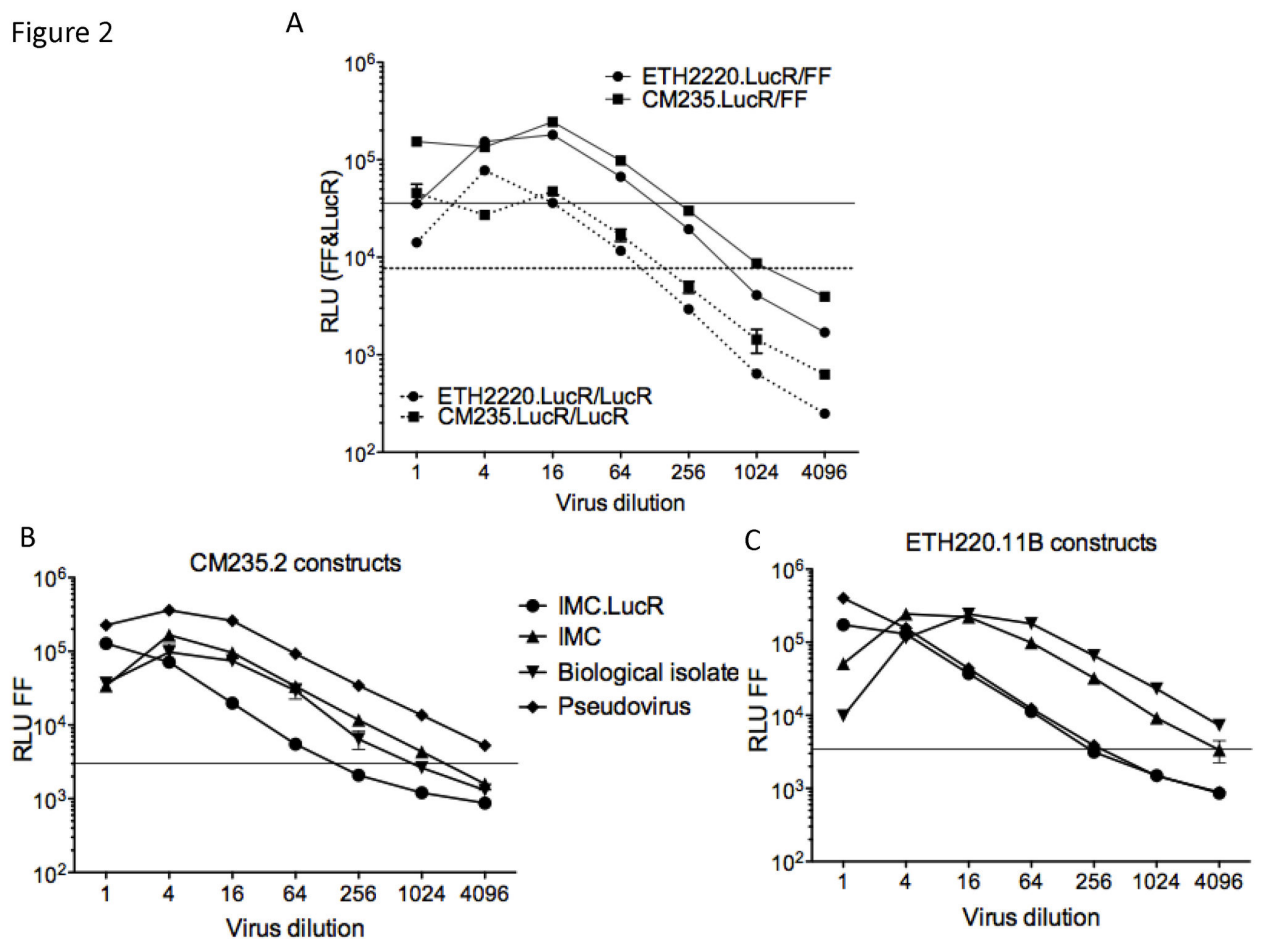
Viral titrations in TZM-bl cells. Titrations of IMC.LucR only, CM235.LucR and ETH2220.LucR (A), of CM235.2 Env-based viruses (B) and of ETH2220.11B Env-based viruses (C). All titrations were performed in duplicate, in a 4-fold dilution format. Firefly luciferase acitivity (RLU FF, bold lines) was measured 48 hours later. For IMC.LucR only (A), Renilla luciferase activity (RLU LucR, dashed lines) was also measured simultaneously in the same wells. The different viral forms expressing Env of CM235.2 (B) and ETH2220.11B (C) were compared: virus stocks of pseudovirus (diamond), biological isolate (inverted triangle), parental IMC (triangle) and IMC.LucR (circle). Horizontal solid and dashed lines indicate the cut-off values for FF and LucR, respectively.

 The functionality of our various IMC.LucR constructs was first tested in TZM-bl cells. This cell line had been engineered to contain a *tat*-inducible firefly (FF) luciferase reporter gene to measure HIV-1 infection [30]. In the same well that contains infected-cells, we could simultaneously monitor both cell-encoded FF and virus-encoded LucR luciferase activities as shown in Figure 2A. For both CM235.LucR and ETH2220.LucR, the plots of FF and LucR RLUs over virus input dilutions had nearly identical linear range and slope (Figure 2A) and gave an equivalent titer (10^4.2^ TCID_50_ for CM235.LucR and ETH2220.LucR). We evaluated the titer of these two newly constructed IMC.LucR along with the four different viral forms expressing CM235.2 and ETH2220.11B Envs. The uncloned primary isolates, pseudoviruses, IMC, and IMC.LucR were tittered on TZM-bl cells. In general, both CM235.LucR and ETH2220.LucR transfection-derived viral stocks were infectious with somewhat lower titers than the other viral forms (Figure 2B and 2C). To compare infectivity, we first determined the p24 concentration of each virus stock, then titered the stocks using a standardized p24 input (Figure S2). All HIV molecular clone-based stocks generated by 293T/17 cell transient transfection showed similar results with no infection observed at a p24 concentration ≤ 100 pg/ml. Inversely, both PBMC-derived biological isolates were still infectious at a p24 concentration of 2 pg/ml. Louder et al. previously reported that molecularly cloned viral stocks exhibit far fewer env trimers per virion when produced by transfection of 293T/17 cells rather than infection of PBMCs [36]. A difference in the number of Env molecules expressed at the surface of the virions may partially explain the higher infectivity obtained with biological isolates. 

**Figure 3:**
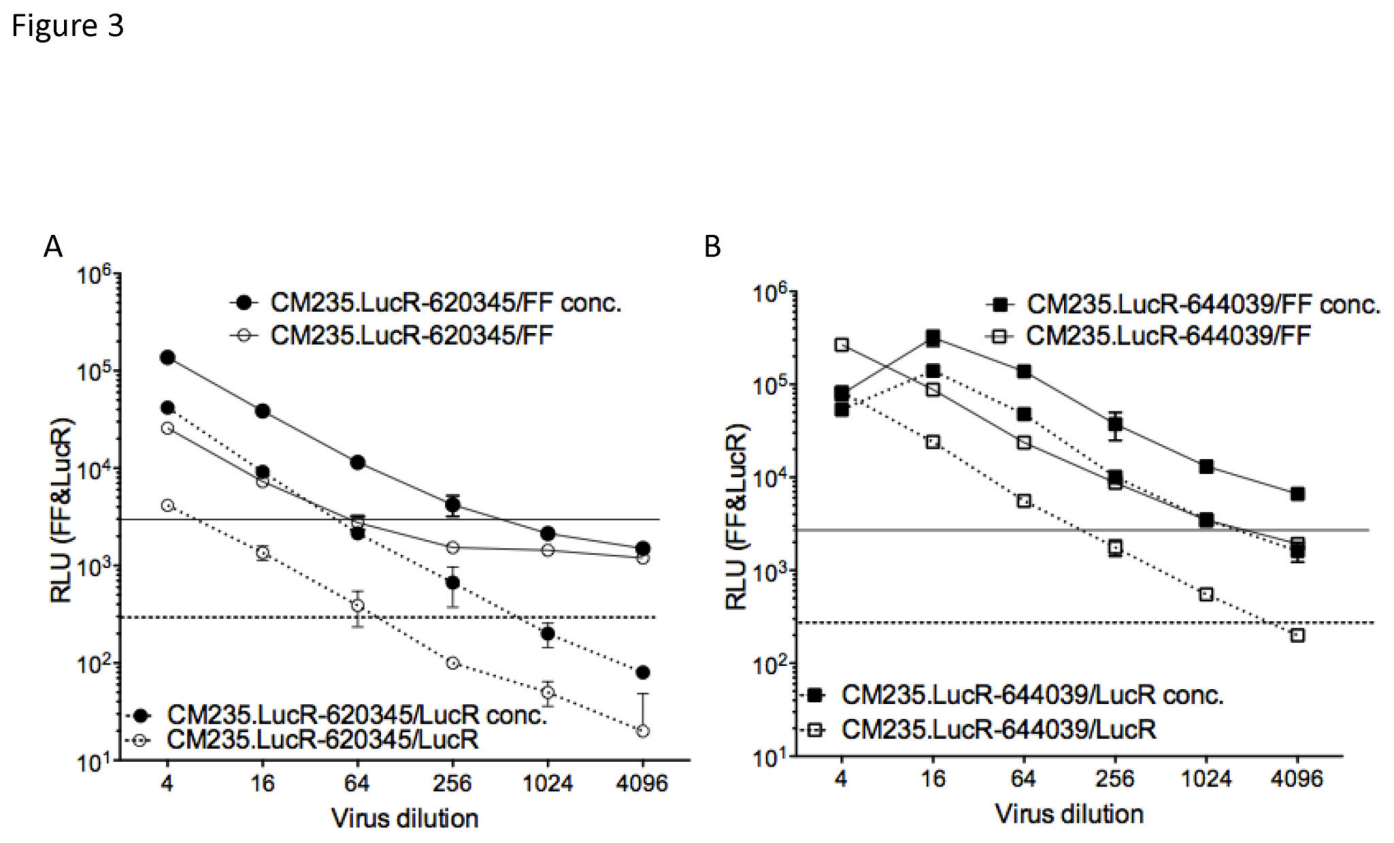
Titrations in TZMbl cells of chimeric IMC.LucR before and after concentration. We measured CM235.LucR-620345 (A) and CM235.LucR-644039 (B) concentrated (open symbols) and original (plain symbols) virus stocks. Titrations were performed in duplicate, in a 4-fold dilution format, in TZM-bl cells. Firefly luciferase acitivity (RLU FF, bold lines) and Renilla luciferase activity (RLU LucR, dashed lines) were measured simultaneously in the same wells, 48 hours later. Horizontal solid and dashed lines indicate the cut-off values for FF and LucR, respectively.

The chimeric IMC.LucR-Env constructs (Table 1) were titered on TZM-bl cells and ranged in titer from 10^2.2^ to 10^5^ TCID_50_. In order to clone the entire *env* DNA sequences (including signal peptide and entire gp41 endodomain) we have generated unique chimeric Tat and Rev proteins that could have an impact on viral replication. We significantly improved low-titer virus stocks through concentration by filtration; as an example, Figures 3A and 3B show the titers of chimeric CM235.LucR-620345 and CM235.LucR-644039 on TZM-bl cells, before and after concentration. For CM235.LucR-620345 (Figure 3A), the titer increased from 10^2.5^ to 10^3.4^ TCID_50_, and for CM235.LucR-644039 (Figure 3B), from 10^4^ to 10^4.8^ TCID_50_. Similar titer values were determined using FF and LucR readouts.

### IMC.LucR infects CD4+T cells

**Figure 4:**
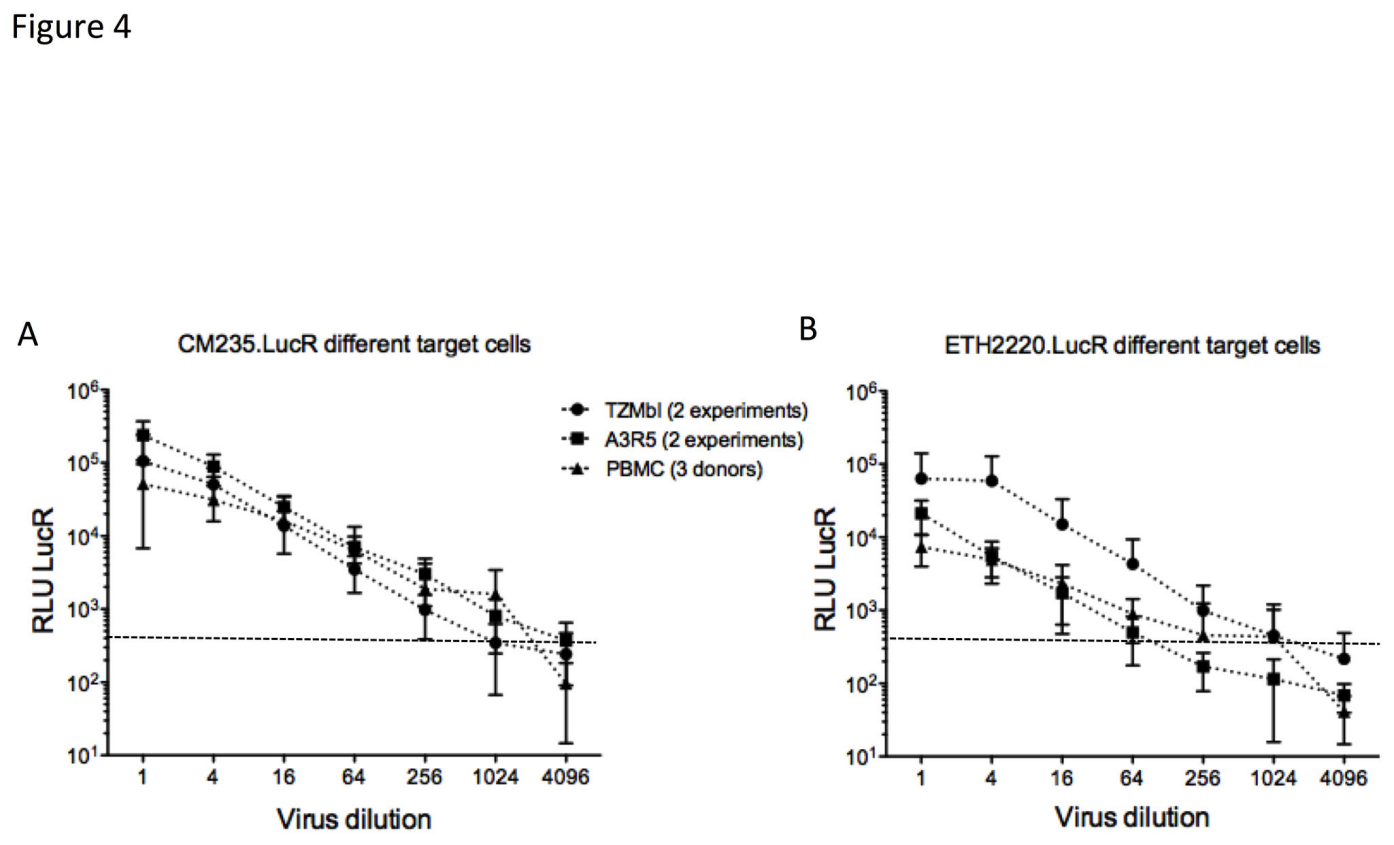
Titrations of CM235.LucR and ETH2220.LucR in different target cells. We also assessed infectivity of CM235.LucR (A) and ETH2220.LucR (B), in the cell lines TZMbl (two independent experiments) and A3R5 (two independent experiments), and in PBMC (three donors). RLU LucR was measured 72 hours after infection (dashed lines). Horizontal lines represent RLU cut off for LucR (dashed) activity.

 The ability of CRF01_AE and subtype C IMC.LucR viruses to infect different target cells was assessed using the LucR output (as measured by RLU). We compared infectivity of CM235.LucR (Figure 4A) and ETH2220.LucR (Figure 4B) using TZM-bl cells, a CD4+T-cell line (A3R5) [24], and PBMC; both viral constructs infected all target cell types and exhibited relatively comparable titers in the different target cells. However, ETH2220.LucR showed lower infectivity in CD4+T cells (A3R5 and PBMC) as compared with epithelial cell line TZMbl (Figure 4B). This difference may be explained in part by the difference in CCR5 and/or CD4 level of expression on the surface of the engineered TZM-bl cells and by Env specificity and the resulting receptor-Env interactions. The high level of CCR5 expressed in the TZM-bl cell line [37] when compared to the two other cell types, may not reflect accurately ETH2220 entry efficiency. We confirmed productive infection and de novo replication of our constructs in infected PBMC, using Zidovudine (AZT), which inhibits the reverse transcriptase and prevents HIV-1 replication, (data not shown). 

### LucR does not affect IMC neutralization sensitivity in the TZM-bl assay

**Figure 5:**
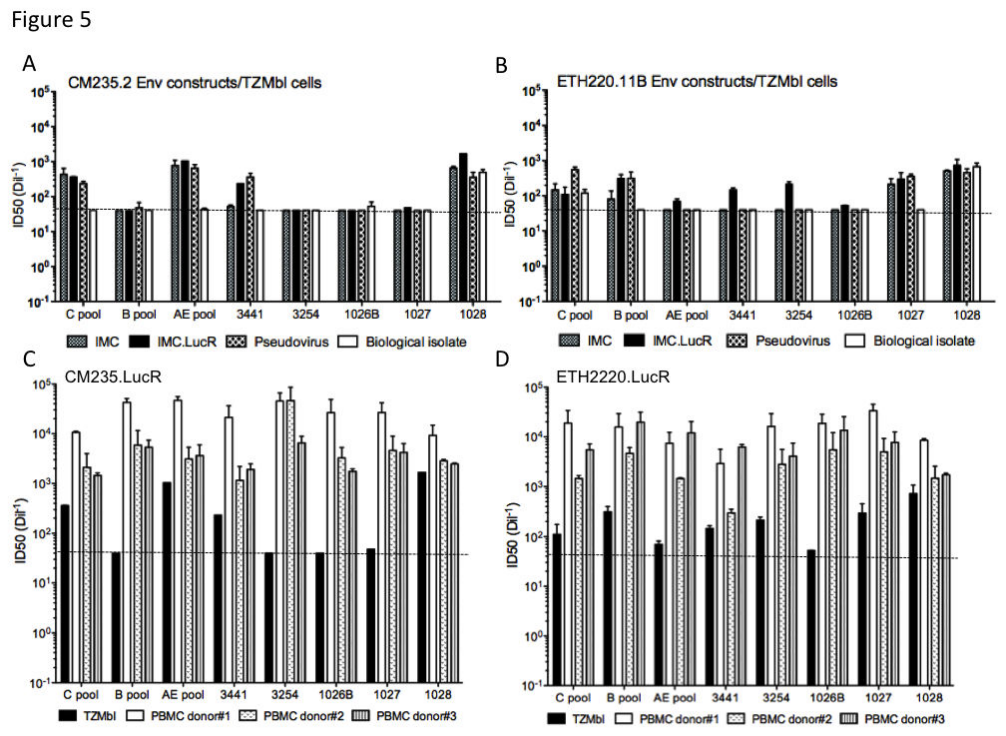
Neutralization profiles CM235.2 and ETH2220.11B Env-based viruses. Inhibition of infection using HIV-1+ sera and multiple viral forms (pseudovirus, biological isolate, original IMC and IMC.LucR) of CM235.2 (A) and ETH2220.11B (B) in the TZM-bl assay, using the cell-line encoded firefly luciferase reporter endpoint. Inhibition of infection using HIV-1+ sera and CM235.LucR (C) and ETH2220.LucR (D) in the TZM-bl and PBMC assay with three different donor PBMC as assay targets, using the IMC-encoded Renilla luciferase reporter endpoint. Values are the reciprocal sera dilution at which RLU was reduced by 50% compared to the level in virus control wells. Horizontal lines represent the threshold of detection (1:40 sera dilution); values at or under the line indicate 50% inhibition was not reached.

 We then confirmed that the insertion of the cassette LucR did not modify the neutralization sensitivity of our parental AE- and C-IMC constructs when compared to the other infectious viral forms (IMC-LucR, biological isolate and pseudovirus) expressing CM235.2 (Figure 5A) and ETH2220.11B (Figure 5B) Envs. Virus stocks were compared using the TZM-bl neutralization assay (with firefly luciferase readout) and a panel of polyclonal sera. We found that the NAb titers of CM235.LucR and ETH2220.LucR were similar to parental IMCs and pseudoviruses with the range in ID50 values not exceeding 3-fold, (average of 2.5-fold and 2.6-fold, respectively for CM235.LucR and 1.7-fold and 1.7-fold, respectively for ETH2220.LucR). CM235 and ETH2220 primary virus isolates were poorly neutralized by most sera.

### IMC.LucR exhibit neutralization sensitivity in PBMC

 Neutralization assays were performed in parallel on TZM-bl cells and PBMC using CM235.LucR (Figure 5C), and ETH2220.LucR (Figure 5D); LucR readout was used to determine the ID50 values. The PBMC assay allows for multiple rounds of viral replication and concurrently measures several mechanisms of antibody-dependent viral inhibition [25,27]. Both CM235.LucR and ETH2220.LucR were found to be sensitive to neutralization mediated by HIV-positive polyclonal plasma; comparable results were obtained using three different donor PBMC as assay targets (Figure 5C & D). Overall, the titers obtained using CD4+ PBMC targets from all three donors were higher than those observed in TZM-bl cells. This observation of lower levels of neutralization sensitivity using TZM-bl target cells has been previously reported [38,39]

### Differences in neutralization sensitivity are observed in the TZM-bl assay when Env is expressed in a subtyped-matched versus–mismatched backbone

 To investigate if the HIV backbone can influence neutralization sensitivity, we first looked at the neutralization profiles of CRF01_AE CM235.2 and subtype C ETH2220.11B Envs when expressed in their native backbones, CM235.LucR and ETH2220.LucR, or in subtype-mismatched backbones, CM235.LucR-ETH2220 and ETH2220.LucR-CM235 (Table 2). Differences in neutralization sensitivity were observed between the original IMC.LucR and chimeric, subtype-mismatched IMC.LucR-Env, with mAbs as well as with individual HIV+ plasma, with six data pairs showing over 3-fold differences, but only one data pair over a 5-fold difference (using plasma 3254). However, for these chronic Envs, the subtype specificity of certain polyclonal pools could be observed no matter which backbone was used, eg. the AE-pool neutralized the AE-Env more potently than the C-Env, and the B pool neutralized the C Env, while showing no cross-neutralization of the AE Env, confirming previous observations of subtype specificities with these pools [35].

**Table 2:** Neutralization profile of parental AE- & C-IMC.LucR and subtype mismatched chimeric IMC.LucR-Env in the TZM-bl assay.

**Env**	**Backbone**	**4E10** ^1^	**sCD4** ^1^	**C pool** ^2^	**B pool** ^2^	**AE pool** ^2^	3441^2^	3254^2^	1026^2^	1027^2^	1028^2^
CM235	CM235	4.2	>25	363	<40	1039	232	<40	<40	48	1674
CM235	ETH2220	5.0	>25	111	<40	1272	647	89	52	147	1136
ETH2220	ETH2220	4.1	14.1	111	313	70	145	213	53	295	726
ETH2220	CM235	2.2	3.6	405	207	<40	<40	<40	77	212	1098

^1^ Mean inhibitory concentration IC50: no neutralization IC50>25 (μg/ml), poor >15, low to medium IC50 5-15 (μg/ml), and good IC50 0-5 (μg/ml).

^2^ 50% inhibitory dilution D50: no neutralization ID50 <40 (Dil^-1^ , poor ID50 40-250 (Dil^-1^ , low to medium 250-1000 (Dil^-1^ , and good >1000 (Dil^-1^

### Subtype-mismatched Env/backbone pairs resulted in greater neutralization sensitivity to some inhibitors

To further investigate this phenomenon, we evaluated additional Env/backbone pairs that were subtype-matched or mismatched, by expressing three subtype C and six CRF01_AE Envs into both CRF01_AE and subtype C IMC.LucR backbones (Table 1). Most of these Envs were isolated very early from patients in Fiebeig stage I/II, and all Envs were thus heterologous to their backbones CM235.LucR and ETH2220.LucR. 

**Figure 6:**
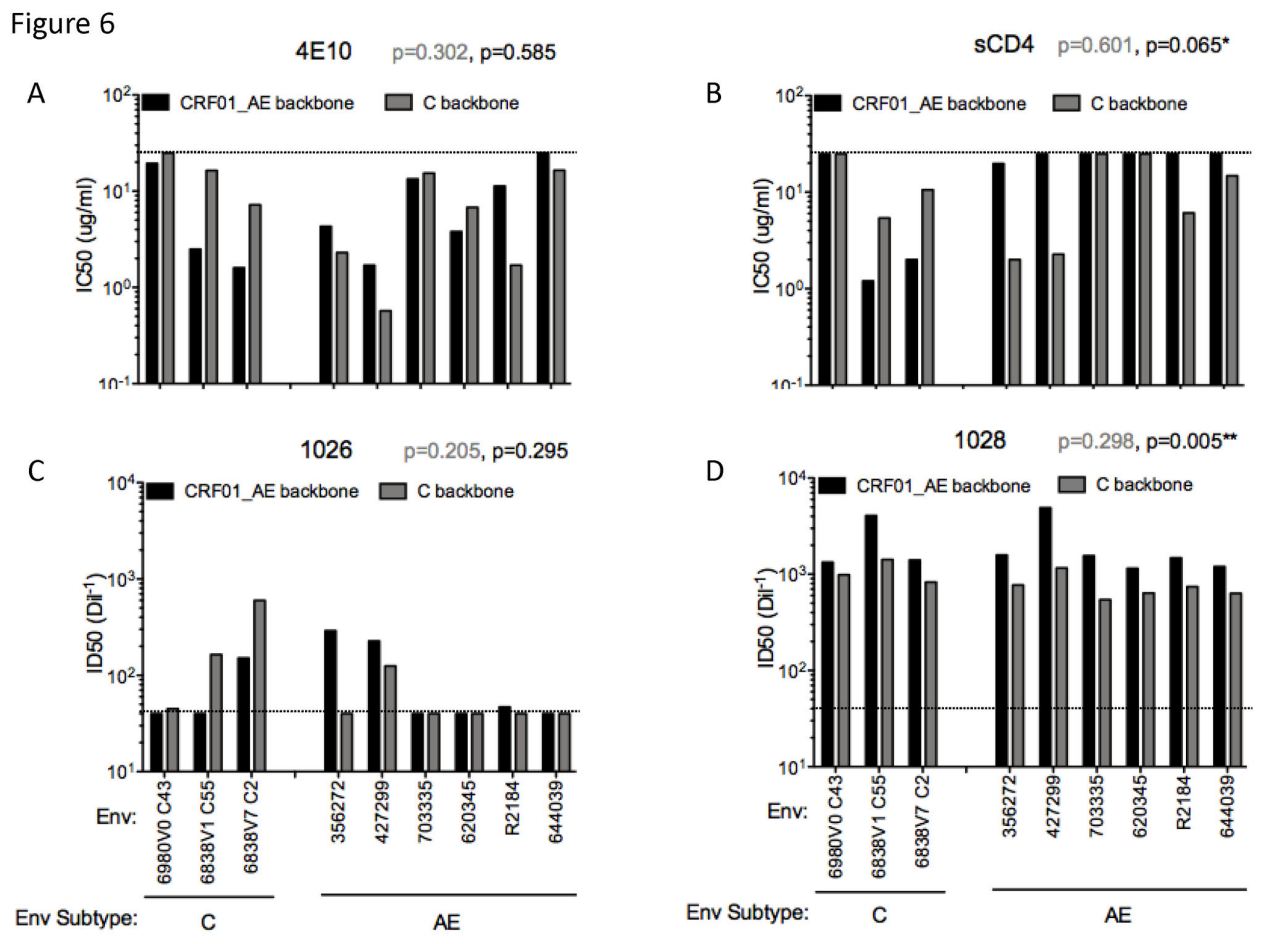
Neutralization sensitivity of Env expressed in subtype-matched vs mismatched backbones. Envs from three subtype C and six CRF01_AE isolates were exchanged into both the subtype C ETH2220.LucR and the CRF01_AE CM235.LucR backbones. Most of the Envs we tested were acute, except for 6838v7 and R2184. Neutralization titers for each stock were generated in TZM-bl cells using (A) 4E10 and (B) sCD4 and polyclonal plasma (C) 1026 and (D) 1028. The Y-axis represents the IC50 (A & B) or ID50 (C & D) values for each Env in both backbones. For statistical analysis, we compare, within Env subtype, neutralization sensitivity by reagent between different backbone constructs (Wilcoxon non-parametric test); p-values for C-envs are written in grey and for AE-envs in black. Horizontal lines represent the cut-off: for (A) and (B), values under the cut-off lines (25 ug/ml) represent neutralization sensitivity, when for (C) and (D) values under the cut-off (dilution 1:40) show resistance to neutralization.

 Neutralization profiles of all IMC.LucR-Env were then determined in TZM-bl cells using 4E10, sCD4 and two individual HIV-positive plasma samples with either low-titer (1026) or high-titer (1028) neutralizing activity (Figure 6A, B, C, and D). Differences in neutralization sensitivity were observed; analysis of this larger data set allowed the effect of backbone on neutralization sensitivity to be visualized. Interestingly, the backbone used to express different Envs seemed to impact sensitivity to inhibition by sCD4 and by the 4E10 NAb. The chimeric IMCs having CM235.LucR or ETH2220.LucR backbones with subtype-mismatched gp160 Envs were more sensitive to inhibition by 4E10 and sCD4. The three subtype C Envs showed greater sensitivity to 4E10 when expressed in the mismatched CRF01_AE backbone (Figure 6A), but the sample size was too small to generate meaningful statistics. The CRF01_AE Envs that were resistant to sCD4-mediated inhibition in CM235.LucR subtype-matched backbone showed a trend toward increased neutralization sensitivity when expressed in the ETH2220.LucR subtype-mismatched backbone (p=0.065).

 Neutralization by more potent plasma (1028) was always greater when the CRF01_AE backbone was used, regardless of Env; this difference was found to be significant for the acute CRF01_AE Envs (p=0.005). However, when using the plasma with low-titer neutralizing activity (1026), greater neutralization was detected when using subtype-matched backbones for both subtype C and CRF01_AE Envs. The NAbs, IgG1b12 and 2F5 were also tested against these viral constructs, but the IMCs were generally resistant to neutralization by these NAbs (as are the matched pseudoviruses), (data not shown).

## Discussion

 Our data show that HIV-1 backbones may impact the neutralization sensitivity of IMC expressing various complete Env-exchanged isogenic recombinants used for neutralization assays. This observation is of importance to HIV vaccine development, as vaccine developers currently rely on measures of breadth and potency of NAb titers in human sera, against genetically diverse panels of reference HIV-1 Envs [20] that have been thus far expressed solely in a subtype B backbone [21–25]. Indeed, such panels have been generated for the pseudovirion TZM-bl assay [21–23], as well as for the newly developed NL-LucR.T2A-Env.ecto approach which can be used in both TZM-bl, non-reporter cell-lines and PBMC-based assays [24,25]. In the NL-LucR.Env.ecto reporter virus approach, the proviral backbone is derived from the laboratory-adapted HIV-1 subtype B strain (NL4-3), generating constructs with all non-env genes and the gp41 endodomain coding sequence of subtype B origin. It has been reported that modifications in the C-terminal tail of gp41, affecting length or amino acid composition, can have marked affects on global Env structure and function, including envelope incorporation, fusogenicity and antibody reactivity [12,16,17]. For example, the mutation of two arginine residues to glutamate in the gp41 endodomain (LLP2) of an HIV-1 provirus substantially reduced Env fusogenicity and altered antibody reactivity and neutralization sensitivity of epitopes located in gp120 and the gp41 ectodomain [17]. Moreover, recent data suggested that CT of gp41 regulates the conformation of Env and epitope exposure at the cell surface [19], supporting the use of full-length gp160 in neutralization assays. 

 Positing that the effect of strain or subtype of the backbone should be carefully considered, we generated non-B IMC (IMC.LucR) with Thai CRF01_AE (CM235.2) and Ethiopian subtype C (ETH2220.11B) strains as HIV-1 backbones into which full-length gp160 can be exchanged. We found that the neutralizing titers for these two IMC.LucR were comparable to titers obtained when using other (non-reporter) HIV-1 backbones to express the cognate Env, with the exception that primary isolate titers were typically lower. These differences may be due to the diversity of the HIV-1 genomes present in uncloned viral stocks, or to the sources of virus stock production (293T/17 cell transfection versus PBMC propagation), which are beyond the scope of this study. To create the IMC –LucR constructs for *env* exchange described here, we generated viral genomes with chimeric *tat* and *rev* genes (Figure 1). It is important to note that this type of chimerization occurs naturally in HIV-1 recombinants found within infected patients. An analysis of unique and circulating recombinants within the Los Alamos database revealed that over 40% of recombinant isolates sequenced from patients in both chronic and acute infection contained recombinant *tat* and *rev* genes (http://www.hiv.lanl.gov/content/sequence/HIV/CRFs/CRFs.html). Thus, the chimeras produced here do not represent a complete deviation from the genetic recombinants that occur in nature.

 To explore the impact of non-*env* genes on Env-mediated neutralization, we exchanged acute subtype C and CRF01_AE gp160 Envs into both parental backbones, CM235.LucR and ETH2220.LucR, and analyzed neutralization profiles of these chimeras in the TZM-bl assay. CRF01_AE Envs, which were resistant to sCD4-mediated inhibition in the CRF01_AE backbone, (CM235.LucR) became sensitive when exchanged into the subtype C backbone, ETH2220.LucR (p=0.065). A similar trend towards increased sensitivity in a subtype mismatched-backbone was observed with 4E10 for both AE and C Envs. Differences observed in 4E10 and sCD4 titers may indicate structural changes in Env imposed by the backbone genes on the membrane proximal external region (MPER) and CD4-binding site of gp120, respectively. Substantial backbone-related differences were also observed in plasma titers (p=0.005 with plasma 1028 for AE Envs) highlighting the importance of the IMC backbone in the analysis of HIV-1 polyclonal sera and thus, vaccinee samples, when using IMC constucts. While our results demonstrate an effect of the HIV-1 backbone on neutralization sensitivity, a greater number of IMCs should be assayed to determine the impact of strain- and subtype- specificity of the backbone on virus immunoreactivity.

 Current HIV-1 vaccines include several viral targets, including Gag, Env and Pol, as both DNA and protein immunogens [40–43]. Antibodies directed against non-Env proteins, such as p17 Gag, have previously been shown to inhibit HIV-1 infection by binding directly to HIV-1 or to HIV-infected cells [24,44–46]. The PBMC assay, which includes both HIV-1 target cells (CD4+ T cells) and anti-HIV-1 effector cells, measures several types of antibody mediated viral inhibition such as ADCC and ADCVI, and may therefore detect non-Env-specific antibody activity more sensitively than cell-line based assays [27]. In the search for relevant vaccine-induced antibody responses, constructs containing the entire genome of genetically relevant HIV-1 strains will need to be compared to strains that are chimeric in backbone/envelope in case-control correlates testing in order to determine which vector system tracks immune responses that correlate with protection from acquisition or disease [47].

## Supporting Information

Figure S1 **Mutation in *vpu* does not affect infectivity.** Infection of TZM-bl cells with (A) CM235.LucR (square) and (B) ETH2220.LucR (circle) that contain (empty symbols) or do not contain (plain symbols) the *BsiWI* restriction site in the *vpu* gene. Both viruses were titrated in duplicate, in a 4-fold dilution format and the firefly luciferase activity was measured 48 hours later (RLU FF).
The horizontal bold line represents RLU FF cut off.
(TIFF)

Figure S2 **p24-standardized titration in TZM-bl cells of CM235.2 Env-based viruses and of ETH2220.11B Env-based viruses.** The different viral forms expressing Env of CM235.2 (A) and ETH2220.11B (B) were compared using a standardized p24 virus input: virus stocks of pseudovirus, biological isolate, parental IMC, and IMC.LucR were assessed for p24 concentration (C and D). Starting with a dose of 25 ng/ml of each virus stock, a 4-fold serial dilution was used to infect TZMbl cells in duplicate. Firefly luciferase activity was measured 48 hours later. The horizontal bold line represents RLU FF cut off.
(TIFF)

Table S1 **List of primers.** The sequences of the forward and reverse primers used to generate the different constructs are listed.
(DOC)
