# Supplementary figures and images for: Impact of HIV-1 Backbone on Neutralization Sensitivity: Neutralization Profiles of Heterologous Envelope Glycoproteins Expressed in Native Subtype C and CRF01_AE Backbone
Source: PLoS One. 2013 Nov 29;8(11):e76104. doi: 10.1371/journal.pone.0076104 (PMC3843658; doi:10.1371/journal.pone.0076104)

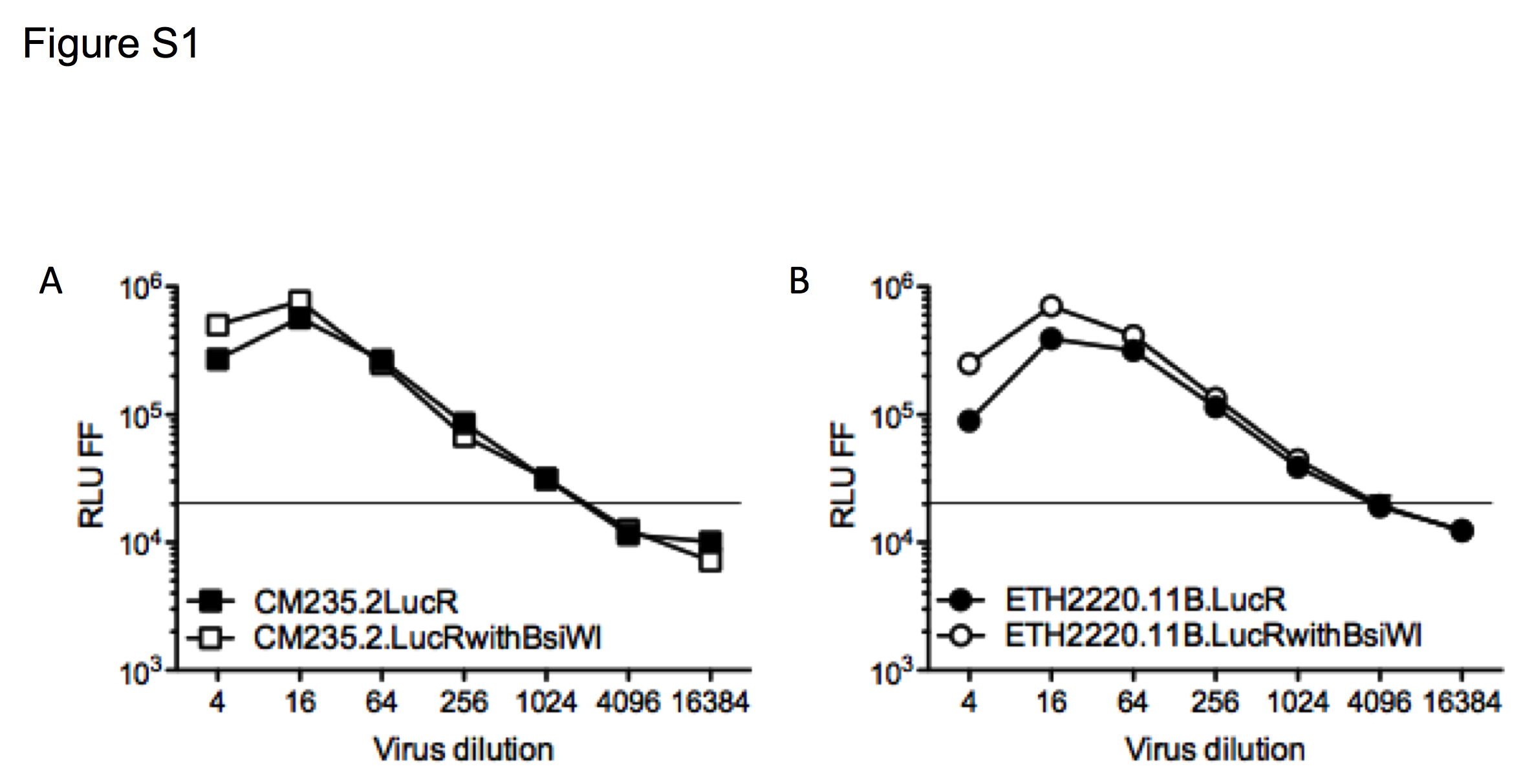

Supplement: Figure S1 — Mutation in vpu does not affect infectivity. Infection of TZM-bl cells with (A) CM235.LucR (square) and (B) ETH2220.LucR (circle) that contain (empty symbols) or do not contain (plain symbols) the BsiWI restriction site in the vpu gene. Both viruses were titrated in duplicate, in a 4-fold dilution format and the firefly luciferase activity was measured 48 hours later (RLU FF). The horizontal bold line represents RLU FF cut off. (TIFF) [file pone.0076104.s001.tiff]

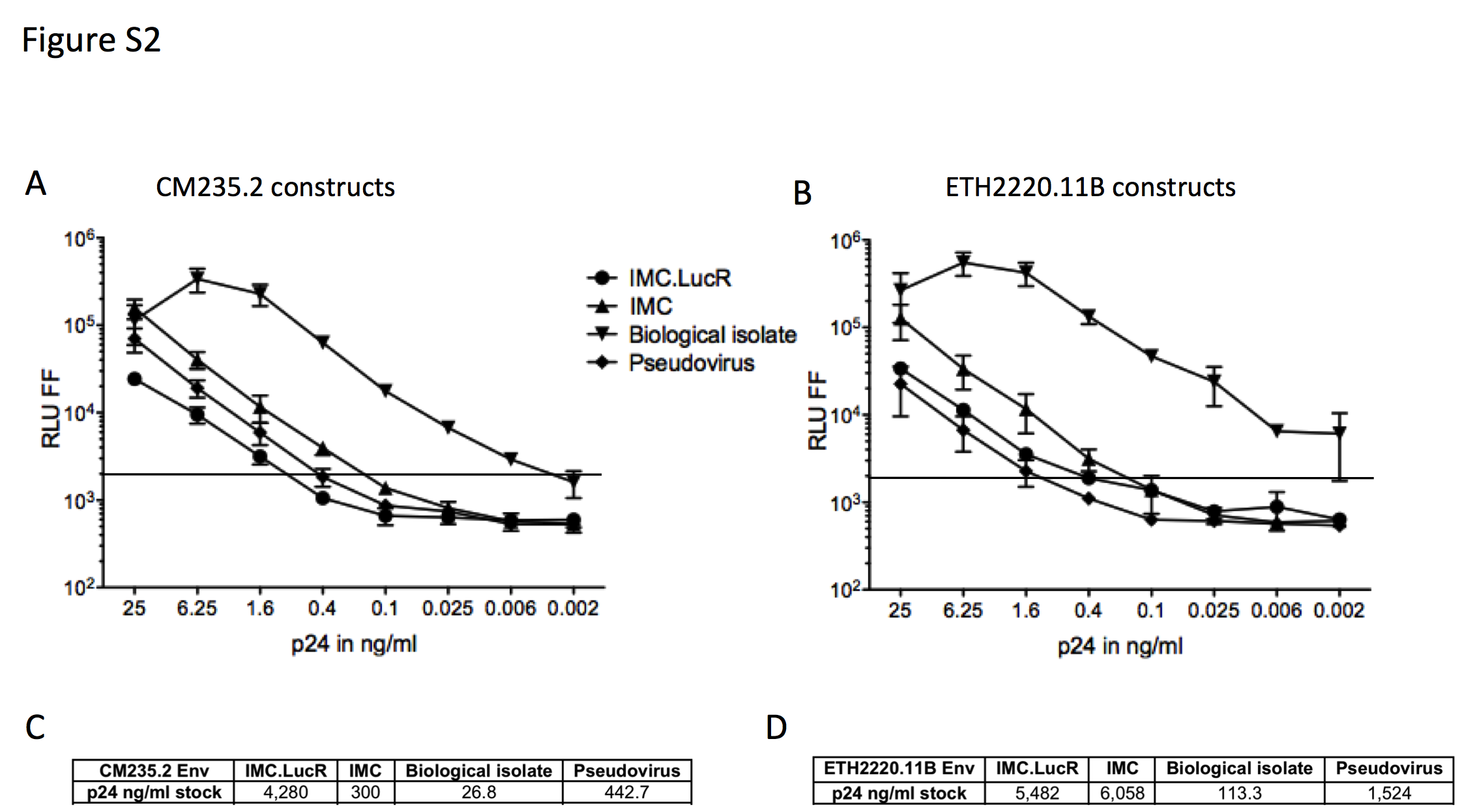

Supplement: Figure S2 — p24-standardized titration in TZM-bl cells of CM235.2 Env-based viruses and of ETH2220.11B Env-based viruses. The different viral forms expressing Env of CM235.2 (A) and ETH2220.11B (B) were compared using a standardized p24 virus input: virus stocks of pseudovirus, biological isolate, parental IMC, and IMC.LucR were assessed for p24 concentration (C and D). Starting with a dose of 25 ng/ml of each virus stock, a 4-fold serial dilution was used to infect TZMbl cells in duplicate. Firefly luciferase activity was measured 48 hours later. The horizontal bold line represents RLU FF cut off. (TIFF) [file pone.0076104.s002.tiff]
