# Supplementary material for: Impact of HIV-1 Backbone on Neutralization Sensitivity: Neutralization Profiles of Heterologous Envelope Glycoproteins Expressed in Native Subtype C and CRF01_AE Backbone
Source: PLoS One. 2013 Nov 29;8(11):e76104. doi: 10.1371/journal.pone.0076104 (PMC3843658; doi:10.1371/journal.pone.0076104)
Supplement: Table S1 — List of primers. The sequences of the forward and reverse primers used to generate the different constructs are listed. (DOC) [file pone.0076104.s003.doc]

Table S1. List of primers used to generate IMC.LucR

| **Name** | **Sequence (5’ to 3’)** | **Sense** |
| --- | --- | --- |
| polk3 | TAAARYTAGCAGGAAGATGGCCAGT | forward |
| LucNheIRV | CGTACACCTTGGAAGCCATGGTCGCTAGCTTATAGCAAAGCCC | reverse |
| LucNheIFW | GGGCTTTGCTATAAGCTAGCGACCATGGCTTCCAAGGTGTACG | forward |
| LucXbaIRV | CCACTTGCCACCCATTCTAGATGGGCCAGGATTC | reverse |
| LucXbaIFW | GAATCCTGGCCCATCTAGAATGGGTGGCAAGTGG | forward |
| pblueKS-r8 | TAAATAAATCCTGGTGTCCCT | reverse |
| AarIENVFW | AGGGATTATGGAAAACAGATGGCAGGTG | forward |
| BglIENVRV | caataaagcttgccttgagggct | reverse |
| A/E_BsiWIvpuFW | GGTTAGTTAAGAGAATTCGCGTACGAGCAGAAGACAGTGG | forward |
| A/E_BsiWIvpuRV | CCACTGTCTTCTGCTCGTACGCGAATTCTCTTAACTAACC | reverse |
| C-BsiWIvpu RV | CCACTGTCTTCTGCTCGTACGCGAATTCTTTTAACTAACC | reverse |
| C_BsiWIvpuFW | GGTTAGTTAAAAGAATTCGCGTACGAGCAGAAGACAGTGG | forward |
| MluI_EFW | CGAAAGGGCTTTGCTATAAACGCGTGACCATGGCTTCCAAGG | forward |
| MluI_ERV | CCTTGGAAGCCATGGTCACGCGTTTATAGCAAAGCCCTTTCG | reverse |
| MluI_CFW | GGAAGCAGCTTTGCAATAAACGCGTGACCATGGCTTCCAAGG | forward |
| MluI_CRV | CCTTGGAAGCCATGGTCACGCGTTTATTGCAAAGCTGCTTCC | reverse |
| EcoRIbalectoFW | GCCATAATAAGAATTCTGCAACAACTG | forward |
| JL70RV | ACCCCATAATAGACTGTGACCCACAA | reverse |
| A/EectoKpnIRV | CCACACAGGTACCCCATAATAAAC | reverse |
| JL70 | TTGTGGGTCACAGTCTATTATGGGGT | forward |
| A/EectoKpnIFW | GTTTATTATGGGGTACCTGTGTGG | forward |
| cladeCectoRV | GCAAAAACTATTCGAAGACCTATCACACCTCCTAC | reverse |
| cladeA/EectoRV | GCAAAAACTATTCGAAGACCTATTAAACCTCCTATTATC | reverse |
| cladeCectoFW | GTAGGAGGTGTGATAGGTCTTCGAATAGTTTTTGC | forward |
| clade A/EectoFW | GATAATAGGAGGTTTAATAGGTCTTCGAATAGTTTTTGC | forward |
| NL43BamHIRV | GCTAAGGATCCGTTCACTAATC | reverse |
